# Corneal ectasia in mothers of Down syndrome children

**DOI:** 10.1038/s41598-021-02035-2

**Published:** 2021-11-17

**Authors:** Hassan Hashemi, Soheila Asgari, Parsa Panahi, Shiva Mehravaran, Akbar Fotouhi, Renato Ambrósio

**Affiliations:** 1 Noor Ophthalmology Research Center, Noor Eye Hospital, No. 96 Esfandiar Blvd., Vali’asr Ave., Tehran, Iran; 2 Student Research Committee, School of Medicine, Iran University of Medical Sciences, Tehran, Iran; 3 School of Computer, Mathematical, and Natural Sciences, Morgan State University, Baltimore, MD USA; 4 Department of Epidemiology and Biostatistics, School of Public Health, Tehran University of Medical Sciences, Tehran, Iran; 5 Rio de Janeiro Corneal Tomography and Biomechanics Study Group, Rio de Janeiro, RJ Brazil; 6 Department of Cornea and Refractive Surgery, Instituto de Olhos Renato Ambrósio, Rio de Janeiro, Brazil; 7 Department of Opthalmology, Federal University of the State of Rio de Janeiro (UNIRIO), Rio de Janeiro, Brazil

**Keywords:** Medical research, Translational research

## Abstract

In this study, corneal findings regarding keratoconus (KC) and early KC among mothers with Down syndrome children (MDS) and a group of age-at-delivery-matched mothers with normal children (MNC) were compared. KC was diagnosed based on the presence of a clinical sign and at least one abnormal tomographic or biomechanical criterion. Early KC was defined as having no clinical sign in the presence of at least one abnormal tomographic or biomechanical criterion. The normal subgroups in each group were compared in terms tomographic and biomechanical parameters. In MDS and MNC, the prevalence rates were 6.5% and 1.6% for KC (P = 0.047), and 30.9% and 14.3% for early KC (P = 0.014), respectively. Comparison between the two normal subgroups showed significant differences in mean index of height asymmetry, irregularity index, anterior asphericity, pentacam random forest index, corneal stiffness parameters at first applanation, deformation amplitude ratios, integrated radius-1 mm, highest concavity deflection amplitude, biomechanical corrected IOP, peak distance, and radius (all P < 0.05). This study showed that MDS are more likely to have KC and also to have thinner, steeper and softer corneas compared to MNC. This results support the need for further work for determining the risk of delivering a child with DS.

## Introduction

Corneal ectatic disorders can lead to loss of vision due to progressive corneal thinning^1^. Clinical signs and results of advanced corneal imaging and biomechanical properties are considered in the diagnosis, classification, and severity evaluation of keratoconus (KC)^2,3^. Although the exact cause of KC is still unknown, previous studies have indicated that it is a multifactorial disease, and combinations of several factors, including genetics and environment, are involved^2–5^. Since genetic factors play an important role in the development of KC, the occurrence of this disease can be associated with other genetic disorders and syndromes^2,6^.

Down syndrome (DS) is a genetic condition in which the affected person has an extra chromosome 21 (known as trisomy 21) and some degree of intellectual disability^7^. According to previous reports on DS samples, the incidence of KC is 0.5–15% among these patients (10–300 times higher than the general population)^6^. The higher incidence of KC in this particular population might be attributed to the prevalence of eye rubbing habits and otherassociated hereditary collagen-related disorders in DS patients^8–10^. Although there are many studies about the prevalence of KC in DS patients, there has been no analytical study investigating the correlation between corneal ectasia in mothers and DS birth. This hypothesis was first proposed by Ambrósio in a case report^3^ on a mother with mild KC who had a DS child. Given that DS is one of the major causes of intellectual disability in the world and can affect many aspects of patients’ lives^11^, identification of its possible correlated factors is very important.

Since KC and DS are both relatively infrequent diseases, a case–control study to look at the association between outcome (DS children) and exposure (KC in mother) backward is preferred to long-term follow up a large group for a cohort study^12^. In this study, visual and corneal abnormalities indicating ectasia among mothers who have given birth to Down syndrome children were compared to an age-at-delivery-matched group of mothers who have given birth to normal children.

## Methods and materials

### Patients and sampling

This comparative study was performed in 2020 at Noor Ophthalmology Hospital in Tehran. The cases were mothers with children with Down syndrome (MDS); they were selected from a study of DS children, whose methodology has already been described^13^. Women in the control group were mothers with normal children (MNC); these were selected from the hospital staff and visitors to match the MDS group in terms of maternal age at delivery. Any case with a history of eye surgery was excluded from the study.

### Examinations

All participants underwent a comprehensive ophthalmic examination using slit-lamp biomicroscopy (Haag-Streit, Koniz, Switzerland). Uncorrected (UDVA) and corrected (CDVA) distance visual acuity assessment was performed using SC-2000 Chart (Nidek Co., Tokyo, Japan) and retinoscopy was done using HEINE BETA 200 with ParaStop (HEINE Optotechnik, Herrsching, Germany).

Corneal tomography was assessed using Pentacam HR (software version 6.08r30, data management version 1.21r43; Oculus Optikgeräte GmbH, Wetzlar, Germany) and corneal biomechanics were measured using Corneal Visualization Scheimpflug Technology (Corvis-ST; software version 6.08r30, data management version 1.5r1902; Oculus Optikgeräte GmbH, Germany). All tests were performed between 8 am and 12 noon by two optometrists (one experienced optometrist for each device).

Imaging with Pentacam was repeated until quality specification “OK” (minimum valid data: 93.0%) was reached. Extracted Pentacam indices included the minimum corneal thickness (MCT), maximum Ambrósio's relational thickness (ART-max), Belin Ambrósio display-total deviation (BAD-D), maximum keratometry in the central 8.0 mm (Kmax), average keratometry in the 3.0 mm zone around the steepest point (Zonal Kmax-3 mm), inferior–superior asymmetryy (I–S value), the anterior radius of curvature centered on the thinnest point (ARC), the posterior radius of curvature centered on the thinnest point (PRC), index of surface variance (ISV), index of vertical asymmetry (IVA), keratoconus index (KI), center keratoconus index (CKI), index of height asymmetry (IHA), index of height decentration (IHD), anterior elevation at the thinnest point from the 8 mm best-fit-sphere (AE-TP_8mmBFS), posterior elevation at the thinnest point from the 8 mm best-fit-sphere (PE-TP_8mmBFS), irregularity index, anterior asphericity (Q-value), posterior Q-value, and pentacam random forest index (PRFI).

Extracted indices from the Corvis-ST included the tomographic biomechanical index (TBI), stiffness parameters at first applanation (SP-A1), deformation amplitude ratio at 1 mm (DA ratio-1 mm), deformation amplitude ratio at 2 mm (DA ratio-2 mm), integrated radius-1 mm, highest concavity deformation amplitude (HC deform. ampl.), highest concavity deflection amplitude (HC deflec. amp.), biomechanical corrected intraocular pressure (bIOP), Peak Distance (PD), and radius.

### Definitions

In both groups, corneas were categorized into KC, “mild or fruste” KC—which can be interpreted as with high susceptibility for ectasia progression, and normal subgroups based on ranges of ectasia indices as follows:

- KC: clinical sign (Fleischer ring, Vogt striae, Munson sign, apical thinning, or Rizutti sign) + at least one abnormal tomographic or biomechanical index (BAD-D > 3.0 standard deviation of mean^14^, ART-max < 339 μm^15^, Kmax > 48.0 diopters (D)^16^, I–S value > 1.9 D^17^, and TBI > 0.79^18^).
- “Mild or fruste” KC: no clinical sign + abnormal tomographic or biomechanical index (BAD-D > 1.6 standard deviation of mean^14^, ART-max < 339 μm^15^, Kmax > 47.2 D^16^, I–S value > 1.4 D^17^, or TBI > 0.39^18^).
- Normal: all others.

### Statistical analysis

Statistical analysis was performed using SPSS version 21 (IBM Corp., Armonk, NY, USA). The sample size was determined 56 cases in each group using n = 2(Z_α/2_ + Z_β_)^2^ × σ^2^/d^2^ where the α = 0.05, β = 0.01, σ = 1.0 D for Zonal Kmax-3 mm, and d = 1.5 D. Bonferroni’s correction was not applied to maintain a power of 99% for comparing indices between groups. In the analysis, assuming that the abnormality would be unilateral or asymmetrically bilateral^19^, both right and lefts eyes were entered in the analysis. Binary generalized estimating equations (GEE) were used to compare the prevalence of KC and early KC between the two groups (MDS and MNC). For normal subgroups of MDS and MNC, linear GEE was used to compare mean values of the indices. In the analysis, fellow-eye correlations were applied using an unstructured correlation matrix.

### Ethical consideration

The Ethics Committee of Tehran University of Medical Sciences approved the study (ID: IR.TUMS.MEDICINE.REC.1399.772). The aims and methods of the study were explained to the participants and written informed consent was obtained. The study adhered to the Helsinki Declaration at all stages.

## Results

In this study, after applying the inclusion and exclusion criteria, 140 participants were enrolled; 136 eyes of 77 MDS cases were compared with 126 eyes of 63 MNC controls. Mean maternal age at birth was 31.34 ± 6.86 (range: 17.0–49.0) years in MDS and 30.63 ± 5.02 (range: 22.0–46.0) years in MNC (P = 0.342). The mean age at the time of examination was 48.81 ± 6.93 in MDS and 48.46 ± 8.35 years (P = 0.713).

KC was diagnosed in 6.5% (5 cases) in the MDS group and 1.6% (1 case) in the MNC group; the prevalence of KC was significantly higher among MDS (P = 0.047). Mild or fruste KC was identified in 30.9% (21 cases) in the MDS group and 14.3% (9 cases) in the MNC group (P = 0.014).

Table 1 summarizes results of the comparisons between the normal MDS subgroup (n = 84 eyes) and the normal MNC subgroup (n = 106 eyes). As presented in this table, mean intergroup differences were statistically significant for IHA (P = 0.010), irregularity index (P = 0.026), anterior Q-value (P = 0.043), PRFI (0.049), SP-A1 (P = 0.048), DA ratio-1 mm (P = 0.001), DA ratio-2 mm (P = 0.003), integrated radius 1-mm (P = 0.015), HC deflec. amp. (P = 0.026), bIOP (P = 0.049), PD (P = 0.008), and radius (P < 0.001).

**Table 1:** Mean ± standard deviation of vision, refraction, tomographic and biomechanical indices in mothers with Down syndrome children (MDS) with normal cornea and mothers with normal children (MNC) with normal cornea.

	MDS	MNC	P-value*
Number of eyes	84	106	
Age at the time of examinations	48.08 ± 6.61	48.93 ± 8.60	
Maternal age at birth	30.74 ± 6.67	30.25 ± 5.11	
**Visual function**			
UDVA (LogMar)	0.21 ± 0.35	0.26 ± 0.35	0.360
CDVA (LogMar)	0.01 ± 0.03	0.01 ± 0.04	0.621
SE (D)	0.01 ± 1.88	0.25 ± 1.58	0.330
Astigmatism (D)	− 0.69 ± 0.58	− 0.69 ± 0.54	0.963
**Tomographic index**			
MCT (µm)	542.40 ± 27.04	543.20 ± 32.05	0.845
BAD-D	0.90 ± 0.60	0.91 ± 0.57	0.877
ART-max	463.78 ± 86.48	461.21 ± 83.92	0.836
Zonal Kmax (D)	44.26 ± 1.55	44.42 ± 1.34	0.460
I–S value (D)	0.13 ± 0.57	0.08 ± 0.54	0.599
ARC (mm)	7.71 ± 0.26	7.69 ± 0.24	0.471
PRC (mm)	6.29 ± 0.26	6.25 ± 0.20	0.232
ISV	14.95 ± 4.82	14.65 ± 4.20	0.643
IVA	0.106 ± 0.046	0.111 ± 0.044	0.420
KI	1.022 ± 0.021	1.019 ± 0.018	0.272
CKI	1.003 ± 0.005	1.003 ± 0.006	0.921
IHA	3.78 ± 3.86	4.72 ± 4.00	**0.010**
IHD	0.009 ± 0.005	0.010 ± 0.005	0.218
AE-TP_8mmBFS	1.83 ± 1.47	1.81 ± 1.55	0.941
PE-TP_8mmBFS	7.40 ± 4.13	7.40 ± 3.25	0.999
Irregularity	0.020 ± 0.007	0.018 ± 0.006	**0.026**
Anterior Q-value	− 0.32 ± 0.12	− 0.28 ± 0.10	**0.043**
Posterior Q-value	− 0.30 ± 0.16	− 0.27 ± 0.14	0.184
PRFI	0.07 ± .10	0.05 ± 0.05	**0.049**
**Biomechanical index**			
TBI	0.14 ± 0.19	0.13 ± 0.17	0.726
SP-A1 (mmHg/mm)	114.67 ± 23.68	117.74 ± 22.28	**0.048**
DA ratio-1 mm (mm)	1.59 ± 0.06	1.57 ± 0.05	**0.001**
DA ratio-2 mm (mm)	4.28 ± 0.50	4.09 ± 0.37	**0.003**
Integrated radius-1 mm (mm)	6.70 ± 0.85	7.02 ± 0.95	**0.015**
HC Deform. Amp (mm)	0.97 ± 0.09	0.96 ± 0.09	0.325
HC Deflec. Amp (mm)	0.84 ± 0.09	0.81 ± 0.12	**0.026**
bIOP (mmHg)	15.36 ± 2.20	16.04 ± 2.48	**0.049**
PD (mm)	4.89 ± 0.28	4.78 ± 0.28	**0.008**
Radius (mm)	8.71 ± 1.02	8.15 ± 1.04	< **0.001**

Significant values are in bold.

*MCT* minimum corneal thickness, *BAD-D* Belin Ambrósio display-total deviation, *ART-max* maximum Ambrósio's relational thickness, *K* keratometry, *I–S* inferior–superior asymmetry, *ARC* anterior radius of curvature, *PRC* posterior radius of curvature, *ISV* index of surface variance, *IVA* index of vertical asymmetry, *KI* keratoconus index, *CKI* central KI, *IHA* index of height asymmetry, *IHD* index of height decentration, *AE-TP_8mmBFS* anterior elevation at the thinnest point considering 8 mm best-fit-sphere, *PE-TP_8mmBFS* posterior elevation at the thinnest point considering 8 mm best-fit-sphere, *PRFI* Pentacam random forest index, *TBI* tomographic biomechanical index, *SP-A1* stiffness parameters at 1st applanation, *DA ratio-1 mm* deformation amplitude ratio at 1 mm, *DA ratio-2 mm* deformation amplitude ratio at 2 mm, *HC Deform. Amp* highest concavity deformation amplitude, *HC Deflec. Amp* highest concavity deflection amplitude, *bIOP* biomechanical intraocular pressure, *PD* peak distance, *UDVA* uncorrected distance visual acuity, *CDVA* corrected distance visual acuity, *SE* spherical equivalent.

## Discussion

This is the first clinical study comparing corneal tomography and biomechanical properties between MDS and MNC. The results indicated that KC and mild, fruste (or subclinical) KC were more prevalent in MDS, which also had thinner, steeper, and softer corneas compared to MNC even in the absence of definitive KC.

KC is a multifactorial disorder that is instigated by a combination of genetic and environmental factors. However, the exact cause is unknown^20^. To date, several studies have shown and confirmed the role of genetics in the pathogenesis of KC^6,20,21^, and several loci on various chromosomes and single nucleotide polymorphisms responsible for KC have been identified^21^. Such evidence suggest genetic heterogeneity and complex pathogenesis for this disease. One of the genes that have been investigated as a candidate gene for KC in several studies is the superoxide dismutase 1 gene (SOD1)^21–23^. This gene encodes a cytoplasmic enzyme that detoxifies superoxide radicals, a form of reactive oxygen species, and thus reduces the level of oxidative stress in cells^21^. Increased levels of oxidative stress in corneal cells appear to be a predisposing factor for KC, so it has been proposed that a mutation in SOD1 increases oxidative stress and is involved in the pathogenesis of ectatic disorders^21^. Nevertheless, there is no consensus on the role of SOD1 in KC, and there are different pieces of evidence about it^20,21^. SOD1 is the only candidate gene for KC located on chromosome 21, and it could explain the association between KC and DS in some way^21^. In 95% of cases, DS occurs when a gamete with two chromosomes 21 (due to nondisjunction of chromosome 21 pairs in meiosis) with a normal gamete produces a zygote cell with three chromosomes 21 (trisomy 21)^24^. The relationship between DS and KC has been investigated and confirmed in numerous studies^25^. Although eye rubbing due to blepharitis is one of the main causes of KC in DS patients, genetic defects in the structure and composition of the cornea of these patients can be another cause of ectatic disorders^8,26^. Thus, the genes responsible for the defect in corneal biomechanical properties, refractive errors, and the development of ectatic diseases such as KC in DS patients may be located on chromosome 21. On the other hand, it is possible that age-related defects in these particular genes make mothers more susceptible to chromosome 21 nondisjunction in meiosis, thereby increasing the chances of DS births. SOD1 is an example of these genes, which have also been linked to KC; there may be other genes yet unidentified. In this regard, the results of this study indicate that abnormal KC indices are more common in MDS than MNC, but these abnormalities point more to mild KC.

The study has limitations, but open a new horizon of research for the associations between KC and the risk of a mother deliver a child with DS. Following the first anecdotal report, this is the first clinical study that comprises sample sizes large enough to detect statistical significant differences between MDS and MNC. The participants were recruited from different sources from Iran, which makes the findings more generalizable. This study is the lack of genetic testing for KC-related genes (e.g. SOD1) in the participants. The rare nature of KC and DS limited the study design to a correlation study, otherwise we would able to conduct their association with a higher power. Conducting a cohort study would have required enrolling a cohort of KC women throughout their childbearing age and monitoring pregnancy outcomes in term of DS birth; instead, we conducted a case–control study with a lower power to test this hypothesis. Despite the lower power, differences between MDS and MNC were statistically and clinically significant.

The current study detected that MDS are more likely to have KC and also to have thinner, steeper and softer corneas compared to MNC. Such findings support the need for further work but are not to be considered at this point for determining the risk of delivering a child with DS. Indeed, multimodal corneal imaging including corneal tomography and corneal biomechanical assessments should be considered in future larger retrospective studies so that a major prospective study can be defined.

## Data Availability

Data supporting the findings in this report are available from the corresponding author (SA) upon reasonable request.
